# Functional Peptides: Comparing Synthetic and Sequence-Engineered Antibiofilm Pharmaceutics

**DOI:** 10.3390/pharmaceutics18040441

**Published:** 2026-04-02

**Authors:** Bilal Aslam, Muhammad Hassan Khalid, Sulaiman F. Aljasir

**Affiliations:** 1 Department of Veterinary Preventive Medicine, College of Veterinary Medicine, Qassim University, Buraydah 51452, Saudi Arabia; b.aslam@qu.edu.sa; 2 Institute of Microbiology, Government College University Faisalabad, Faisalabad 38000, Pakistan; thehassanmughal@gmail.com

**Keywords:** antimicrobial resistance, antimicrobial peptides (AMPs), biofilm-associated infections, synthetic peptides, engineered peptides

## Abstract

Biofilm formation is a complex phenomenon employed by microbes to counteract antimicrobials. Biofilm-associated infections are a challenging threat to modern medicine. Antimicrobial peptides (AMPs) are recognized as some of the most promising therapeutics to tackle biofilm-producing and multidrug-resistant (MDR) pathogens. However, stability, toxicity, and potency are key issues in the case of naturally occurring AMPs. Next-generation antibiofilm tools, such as synthetic or engineered AMPs, have emerged as a potent therapeutic choice. Synthetic peptides offer structural simplicity, versatility for chemical modification, and increased stability, which makes them capable of effectively disrupting both the biofilm matrix and the bacterial membrane. For engineered peptides, rational sequence modification, hybridization, and computational design are used to overcome limitations related to selectivity, biofilm-specific targeting and regulatory pathway modulation. This review provides a critical evaluation of synthetic and engineered AMPs from various perspectives, such as design strategies, antibiofilm action mechanisms, therapeutic performance, and translational potential. This study sheds light on current advances and emerging technologies, including AI-guided peptide optimization and multifunctional peptide platforms, and thereby sets the stage for the rational development of peptide-based therapeutics aimed at overcoming biofilm-mediated antimicrobial resistance (AMR).

## 1. Introduction

Biofilm-mediated antimicrobial resistance is one of the leading global health threats. Its emergence has been driven by several factors, including the altered metabolism of microbes, greater drug degradation in biofilm matrices, and lower drug effectiveness [1]. Biofilm-producing pathogens, such as *Pseudomonas aeruginosa*, MRSA, *E. coli*, and *Candida* spp., are responsible for infections that are difficult to treat, with limited therapeutic options.

**Figure 1:**
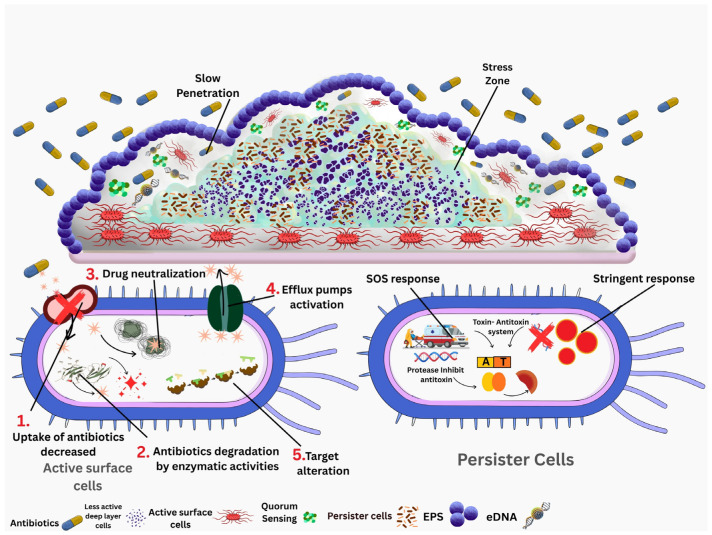
Antimicrobial tolerance mechanisms that are linked to biofilms. The extracellular matrix serves as a barrier to drug penetration, forming metabolic gradients that result in active surface cells and the dormancy of persisters. (1) Surface cells use reduced uptake, (2) enzymatic degradation, (3) neutralization, (4) efflux activation, and (5) target alteration, whereas persister cells survive due to (6) stringent and (7) SOS responses, as well as toxin–antitoxin systems, which together render biofilms stronger and thus cause treatment to fail [5].

Biofilms are a worldwide threat, accounting for 65–80% of all severe and chronic nosocomial diseases, resulting in extended hospital stays and a significant economic impact on healthcare organizations [2]. Biofilms resist traditional antibiotics primarily because their dense matrix physically blocks the penetration of drugs; see Figure 1. This is combined with the development of stress-tolerant persister cells and slowed metabolic activity, especially in deeper layers, which collectively shield microorganisms from the effects of these traditional antimicrobial agents [3]. The drawbacks of traditional antibiotics with regard to biofilms have motivated the scientific community to look for new substances with antimicrobial properties that are effective and non-toxic [4].

Antimicrobial peptides (AMPs) are considered a nature-derived, cost-effective means of eradicating drug-resistant infectious microbes. Natural AMPs display an extensive spectrum of activity against Gram-negative and Gram-positive bacteria, fungi, and viruses, with each one utilizing a different mechanism of action [6]. Nevertheless, their use has some limitations; the latest research shows that utilizing natural AMPs to combat microbes faces challenges such as attack by proteases, shortened life spans, and toxicity to cells [7].

Highly potent engineered antimicrobial peptides resolve such practical concerns. Moreover, engineered antimicrobial peptides (AMPs) have greater biomedical potential than natural ones since their rational design strategies selectively increase their antimicrobial potency and stability while systematically reducing host toxicity. This targeted optimization, achieved through the modification of the structure and residue composition (e.g., stabilizing helices), expands the therapeutic properties of AMPs and enables them to overcome bacterial resistance, demonstrating sufficient effectiveness in long-term clinical applications [8].

**Figure 2:**
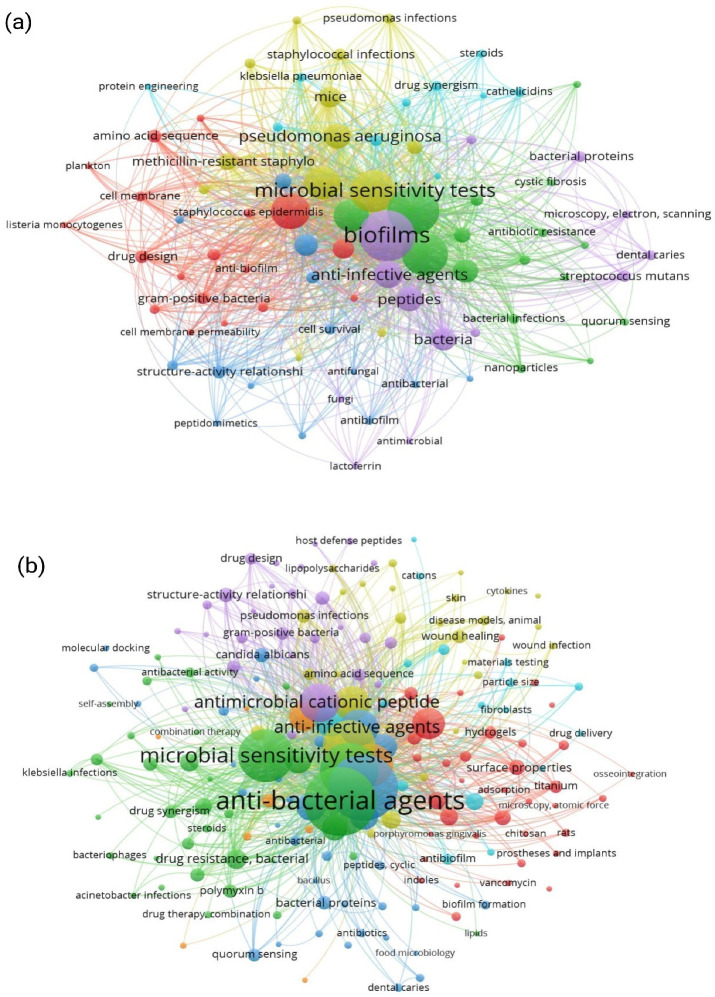
Bibliometric data were retrieved from the PubMed database (2015–2025) using the queries “synthetic antimicrobial peptides AND antibiofilm” and “engineered antimicrobial peptides AND antibiofilm”. Keyword co-occurrence networks were generated and visualized. (**a**) Synthetic antimicrobial peptides emphasize biofilm inhibition and mechanistic antibacterial pathways; (**b**) engineered antimicrobial peptides encompass translational aspects including biomaterials, drug delivery, and implant- and wound-associated infection control.

Substantial experimentation is still ongoing to discover the full potential of AMPs and the shift to engineered AMPs (Figure 2). Moreover, a few key knowledge gaps need to be filled to obtain a systematic, comparative framework, which is essential for the rational design and commercialization of future antibiofilm therapeutics. Likewise, thorough scrutinization of the basic trade-offs between synthetic and engineered AMPs in terms of their mechanistic effectiveness, production costs, and biomedical utility is crucial. This review presents detailed insights into and a practical assessment of synthetic and engineered AMPs, from design to modes of action and from therapeutic potency to a translational perspective.

## 2. Biofilm Resilience and the Case for Peptide Therapy

**Figure 3:**
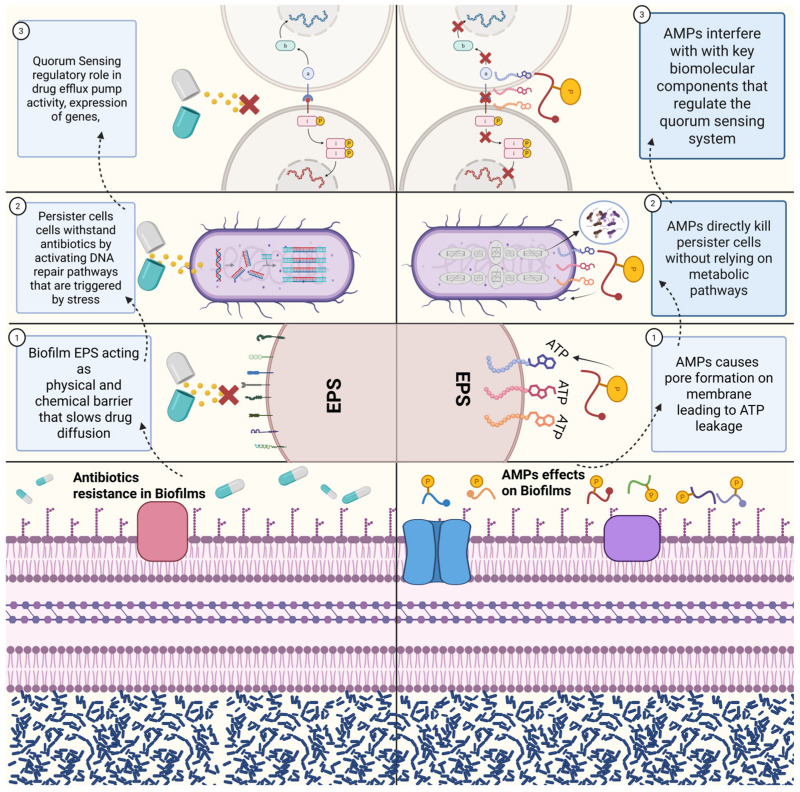
Comparison of the reactions of biofilms to antibiotics and AMPs. Although antibiotics are impeded by the EPS layer, signaling control, and stress responses mediated by persister cells, AMPs circumvent metabolic defenses by attacking membranes, disrupting signaling, and killing persister cells directly.

Microbial biofilms contain an extracellular matrix, which is a very densely packed assembly of polysaccharides, proteins, lipids, and extracellular DNA (eDNA). This characteristic of biofilms plays a very important role in antimicrobial resistance [9]. A broad spectrum of conventional antibiotics, such as azoles, do not achieve the desired therapeutic levels because of their very low penetration through the layer of extracellular polymeric substances (EPSs) [10]. As shown in Figure 3, these antimicrobial peptides are more potent than antibiotics in biofilms because they directly disrupt the cell membrane architecture, thereby creating pores that result in the rapid leakage of ATP and ultimately cell death. Membrane collapse also weakens and degrades the EPS matrix, which in turn leads to the breakdown of the biofilm structure; this is in contrast to antibiotics, which are only capable of diffusing or acting on slower-growing cells. AMPs reduce the biofilm’s physical strength, which gives them an advantage [11]. The resistance of biofilms against antimicrobials is associated with the presence of silent persister cells, which are resistant to conventional antibiotics. These cells resist antibiotics by overcoming stress-induced DNA repair pathways. This process helps to protect them from DNA damage and keeps them active. Consequently, they can withstand regular antibiotics, which are effective only against growing bacteria [12]. Unlike traditional antibiotics, AMPs are capable of eliminating persister cells as they do not depend on any metabolic activity or replication. Instead, they act by destroying the cell membrane, which results in a quick outflow of ions and ATP, thus killing the persistent cells even if they are inactive. This type of action, which is not affected by metabolism, offers AMPs a significant advantage over the conventional antibiotics [13]. Additionally, an equally significant aspect is quorum sensing, which is different among the various microorganisms but still contributes greatly to antimicrobial resistance. Quorum sensing regulates many cellular processes in microbes, including gene expression, toxin production, and extracellular polysaccharide production. It is also involved in the regulation of the drug efflux pump and microbial biofilm formation [14]. Antimicrobial peptides (AMPs) target crucial molecules that control the quorum sensing system and increase the potency of antimicrobial action to levels that even surpass regular antibiotics [15].

## 3. Sequence–Structure–Function Relationships in Antibiofilm Peptides

The antimicrobial strength of several peptides increases when lysine is replaced with arginine, because the guanidinium group of arginine creates better interactions with bacterial membranes [16]. Lysine provides the essential positive charge for initial electrostatic attraction to the negatively charged EPS matrix and bacterial surface. However, arginine uses its guanidinium group to create stronger bidentate hydrogen bonds, which enable the peptide to cross-link with EPS components and penetrate deeper into bacterial membranes and their protective biofilms [17]. The placement of positive charges along a peptide strongly influences how it attaches to and disrupts bacterial membranes. When these charges are strategically clustered, they can enhance antibacterial activity, which minimizes damage to the cells, resulting in improved selectivity [18]. Another important factor is that the antimicrobial peptides use their amphiphilic properties to arrange their hydrophobic and hydrophilic parts, which enables them to create strong electrostatic bonds with biofilm materials while they enter bacterial membranes to disrupt membrane stability [19,20]. Moreover, α-helical structures enable peptides to form amphipathic conformations that insert into lipid bilayers, promoting pore formation or membrane thinning that disrupts cellular integrity. In contrast, β-sheet structures often arise through peptide self-assembly into ordered aggregates or fibrillar architectures that destabilize membranes by forming stable, structured pore-like assemblies [21]. Tryptophan also enhances antimicrobial peptide membrane activity because it binds strongly to lipid–water interfaces. The compound’s large aromatic side chain establishes permanent binding to membrane phospholipids through hydrophobic interactions and hydrogen bond formation, which contribute to the deeper penetration into bacterial membranes [22]. Sequence scrambling studies demonstrate that antimicrobial activity depends not only on amino acid composition but also on the precise order of residues within the peptide. The sequence rearrangement creates a different pattern of amphipathic organization, which decreases membrane interactions despite maintaining constant overall charge and hydrophobicity. This highlights that residue positioning is critical for maintaining the structural alignment required for effective membrane targeting and disruption [23].

## 4. Natural AMPs: Foundational but Insufficient

Natural antimicrobial peptides (AMPs) are promising candidates for the development of a new and effective class of antimicrobial agents to combat antibiotic-resistant pathogens [24]. Natural antimicrobial peptides are defensive molecules that occur in nature and are found in fungi, bacteria, plants, and animals. They are characterized by diverse structural properties and play a critical role as the first line of defense against microbial invaders [25]. Antimicrobial peptides are typically distinguished by their source, the types of amino acids they contain, their physical properties, and their roles. Antimicrobial peptides have a variety of lengths and amino acid compositions. The biological activity of AMPs depends on certain important structural characteristics, such as the helical form, the electric charge, the degree of hydrophobicity, and their total amphiphilic property [26,27].

### Natural AMPs: Microbial-Based

The last few years have seen a surge in the popularity of antimicrobial peptides from various microorganisms due to better insights into the interactions between host and microbes that preserve microbiome equilibrium. Microbial AMP is a new source of substances that can effectively kill off not only harmful bacteria but also multidrug-resistant strains [28]. Bacterial-based antimicrobial peptides (AMPs) are proven to be more effective than other natural AMPs derived from plants or animals, because they are regularly involved in fighting against a variety of bacterial strains [29]. Their lower toxicity, greater resistance to heat, and ability to selectively target the desired organisms make these compounds novel for the development of new types of antimicrobial agents, which can be helpful in clinical therapy, food safety, and agricultural sectors [30]. However, the bacterial-derived AMPs have a significant drawback in that their exact mechanism of biofilm inhibition is still not fully understood. AMPs are indeed very promising, but further research is required to fully realize their potential in the medical field [31]. Furthermore, the strong potential of these AMPs to overcome biofilms is still limited by EPS barriers, biofilm heterogeneity, persistent cells, and the existing limited mechanistic understanding, especially in mature biofilms [32]. The antibiofilm peptides synthesized from *B. subtilis* 6D1 demonstrate these limitations; these peptides inhibit *S. aureus* biofilm formation and modulate gene expression, but the mechanism is not fully understood because the binding affinity and specificity of individual surfactin isoforms to the AgrC receptor, as well as their competition with native *S. aureus* signaling peptides, remain unknown [33]. The major types of antimicrobial peptides (AMPs) derived from bacteria include bacteriocins, microcins, lantibiotics, lipopeptides, thiocins/thiazole-containing peptides, sactipeptides, glycocins, amphipathic α-helical peptides, cyclopeptides/cyclic peptides, and non-ribosomal peptides (NRPs) [34]. The key limitations of these bacterial-derived antimicrobial peptides in combating biofilms are mentioned in (Table 1).

On the other hand, fungi-based AMPs are gaining popularity because of their potential bioactive characteristics, despite being less frequently discussed in the literature [46]. These AMPs have demonstrated significant antibiofilm activity against numerous pathogenic microbes [47]. The primary antibiofilm characteristics of fungal-derived AMPs include detergent-like biosurfactant activity, which plays a crucial role in disrupting the integrity of the cell membrane and killing fungal cells. Its surface-active properties also weaken and physically dislodge the protective extracellular matrix (ECM), thereby eradicating mature biofilms [48]. Most of the AMPs are positively charged and are attracted to the negative fungal membrane, causing cell death via shrinkage and ROS generation. However, their efficacy against mature biofilms is limited, because the extracellular matrix (ECM) traps and inactivates these peptides [49]. Moreover, these fungal-mediated proteins/peptides attach to sessile cells through intracellular uptake, where they trigger ROS accumulation and lead to cell death by apoptosis. Their limited efficacy is demonstrated by the adaptive response of some fungal strains, which can resume growth after initial exposure to these peptides [50]. Some key limitations of these fungal-derived antimicrobial peptides in combating biofilms are mentioned in (Table 2).

Algae are an underexplored yet promising source of such antimicrobial peptides, with many algal AMPs displaying cyclic structures stabilized by disulfide bonds that enhance activity and selectivity. The presence of hydrophobic aromatic residues further contributes to improved antibacterial specificity and potency [56]. Though AMPs from microalgae have huge potential, only a small number have been discovered and characterized. They are usually produced using enzyme-based hydrolysis methods, which break down complex protein structures in algal biomass into small bioactive molecules or peptides [57]. Antimicrobial peptides derived from algae have a great potential for use as antimicrobials, but their mechanisms of action are poorly understood and characterized in an inconsistent manner, which not only limits basic research but also rational optimization and clinical translation. Therefore, the insufficient understanding and inconsistent characterization are the main barriers to the use of algae-derived antimicrobial peptides in the clinical field [58]. In view of the aforesaid limitations to natural AMPs, synthetic and engineered AMPs can be designed with better stability and activity [59].

## 5. Synthetic AMPs: Chemically Designed Peptides with Targeted Antibiofilm Activity

The manufacturing of synthetic antimicrobial peptides (SAMPs) has two main objectives: first, to distinguish the most potent antimicrobial agents; second, to cut off the parts of the sequence that are not only toxic but also easily digestible by proteases [60]. As a result, there are multiple free web-based tools available to forecast and evaluate synthetic antimicrobial peptides (SAMPs). The user can search for potential SAMPs through any protein sequence provided. By using such methods, new peptides with possibly antimicrobial and antibiofilm activities can be identified and rationally designed more efficiently [61].

### 5.1. Designing Synthetic Peptides

**Figure 4:**
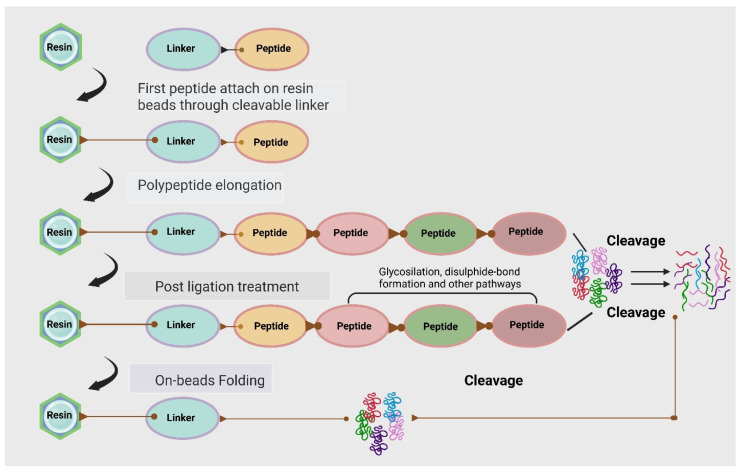
Principle of solid-phase peptide synthesis (SPPS), illustrating stepwise peptide chain assembly on a resin support, followed by post-synthetic modifications, folding, and final cleavage to yield the mature peptide [65].

A multitude of methods are available for the design and synthesis of synthetic peptides. These methods serve to improve the peptides’ antimicrobial and antibiofilm properties, and each of them is classified according to the degree of effectiveness. Ultrashort synthetic AMPs (USAMPs) are designed to be significantly augmented by motifs comprising positively charged amino acids, an overall positive electrostatic charge, and aromatic side chains for enhanced antimicrobial activity [62]. Solid-phase peptide synthesis (SPPS) is the most advantageous technique for preparing synthetic peptides [63]. It relies on sequential amino acid coupling on a resin-bound growing chain (Figure 4), where iterative deprotection and coupling cycles enable controlled peptide elongation while impurities are removed by simple washing [64].

Techniques like fluorenylmethyloxycarbonyl (Fmoc)-based SPPS [66], tert-butyloxycarbonyl (Boc)-based SPPS [67], Solution-Phase Peptide Synthesis (LPPS) [68], Microwave-Assisted Peptide Synthesis (MA-SPPS) [69], and many others have been employed for SAMPs synthesis and design, as shown in (Table 3). Recently, synthetic peptides that incorporate d-amino acids have garnered interest as innovative functional molecules [70]. In one previous study [71], the synthetic antimicrobial peptide K-1dF was derived from Feleucin-K3 by replacing its first phenylalanine with D-phenylalanine. This modification enhances the peptide’s stability against proteases, salt tolerance, and serum stability, while maintaining strong antimicrobial activity against both drug-sensitive and multidrug-resistant *Acinetobacter baumannii*. Synthetic cyclic peptides are also gaining attention in this era due to their remarkable antibacterial and antibiofilm activities [72]. In a previous study [73], lactam-bridged peptide 3 was synthesized by utilizing Fmoc-based ultrasonication-assisted solid-phase peptide strategy (US-SPPS). It showed tremendous antifungal biofilm activity by interfering with surface attachment and forming aggregates on the fungal cell surface. They partially permeabilize membranes without forming full pores, disrupt membrane integrity through a carpet-like mechanism, and adopt amphipathic α-helical structures upon interacting with membranes. Aggregation on the cell surface, helical structure formation, and interaction of hydrophobic and aromatic residues collectively prevent biofilm formation and reduce fungal viability.

### 5.2. Key Design Parameters Influencing Activity

Synthetic antimicrobial peptides perform their best when between 10 and 30 amino acids in length. This length enables them to create stable amphipathic structures that bind effectively to bacterial membranes. Peptides shorter than this may lack structural stability, while excessively long sequences can increase cytotoxicity, production cost, and susceptibility to degradation [74,75]. These peptides show peak performance at positive charge levels between +2 and +9 because this range creates a strong electrostatic attraction toward the negatively charged bacterial membranes [76]. The optimal hydrophobicity for synthetic antimicrobial peptides lies between 30 and 50 percent hydrophobic residues because this range enables effective membrane insertion while maintaining their ability to target specific pathogens [77]. The cyclization process improves antimicrobial peptide performance because it makes peptides more rigid and protects them from being broken down by enzymes, which results in better stability and membrane binding abilities. However, its true impact on potency should be evaluated by comparing cyclic and linear versions with identical sequences under the same experimental conditions, as sequence modifications and testing variations can otherwise confound results [78]. Furthermore, PEGylation modifies antimicrobial peptides through the synthetic bonding of polyethylene glycol chains, which leads to decreased nonselective attachment to host cells and enhanced drug absorption through better water solubility and prolonged drug activity. PEG functions as a hydrophobic area shield that changes how deep materials enter the membrane, which decreases toxic effects while enabling specific binding to bacterial membranes [79,80]. Lipidation is another important phenomenon that improves antimicrobial peptide function by adding fatty acid chains, which create stronger hydrophobic interactions that enable better binding to bacterial membranes, resulting in increased membrane disruption. The process achieves its most effective results when the acyl chain length reaches its optimal measurement because long chains lead to peptide self-assembly, which decreases membrane selectivity and increases toxicity [81].

**Table 3:** Classification of peptides based on design principles, functional objectives, defining features, and commonly employed synthesis strategies.

Peptide Class	Design Strategy/Principle	Key Features/Purpose	Typical Synthesis Technique	Examples
Ultra-short HDAMPs (His-derived antimicrobial peptides)	His N(π)/N(τ) tailoring, Arg tuning, hydrophobic tail optimization	Dual-action, anti-MRSA, LPS neutralization, low hemolysis, protease-stable	SPPS; His dialkylation	HDAMP-1, -3, -5, -6 [82]
Ultra-short Synthetic AMPs (USAMPs)	Rational design; membrane-active; peptide–peptide synergy	Synergistic antibacterial, anti-MRSA, anti-ESBL *E. coli*, low MIC, low hemolysis, reduced resistance	Solid-phase peptide synthesis (SPPS)	WW-185, WOW [83]
Ultra-short Synthetic AMPs (USAMPs)	Length minimization, Arg/Trp positioning; charge–hydrophobicity balance	Broad-spectrum; anti-MRSA, anti-*P. aeruginosa*; anti-*C. albicans*; membrane disruption; low toxicity, cost-effective	Solid-phase peptide synthesis (SPPS)	IRIRIRIR-NH_2_ (18), Pac-525 (28), P9-4 [84]
Ultrashort Arg-Trp-Based Synthetic Antimicrobial Peptides	Multivalency; Arg/Trp-only composition; scaffold-based display	Anti-MRSA; anti-*A. baumannii*, ultrashort peptides, synergistic multivalency, low hemolysis	SPPS; CuAAC “click” chemistry; microwave-assisted synthesis	Trivalent Arg–Trp dendrimers (di- and tripeptide constructs) [82]
D-amino-acid-substituted Synthetic AMP	Full D-amino acid substitution, chirality inversion, stability enhancement	Anti-MDR, membrane disruption, protease-resistant, low hemolysis, retained activity	Solid-phase peptide synthesis (SPPS)	D-MPI (D-Polybia-MPI) [85]
Short Synthetic peptide (NCAA-modified)	lipidation (N- vs. C-terminus), protease resistance	Anti-MDR membrane disruption	SPPS (Fmoc/tBu), DIC/Oxyma; TFA/TIS/H_2_O cleavage	NCAA-lipopeptide analogs [86]
D-amino-acid-modified Synthetic AMP	Full/partial D-amino acid substitution, chirality inversion	Protease-resistant, membrane disruption, anti-MDR, improved in vivo efficacy; reduced hemolysis	Solid-phase peptide synthesis (SPPS)	D-CP, D-lysine-modified polybia-CP [87]
Self-assembling Synthetic AMP (Enantiomeric)	L/D enantiomer comparison, supramolecular self-assembly	Nanoribbon formation, pH-dependent assembly, enhanced activity (D-form), protease-independent potency	Solid-phase peptide synthesis (SPPS)	L-GL13K, D-GL13K [88]
Fatty-acid-conjugated D-AMPs (Lipo-AMPs)	D-amino acid substitution, side-chain lipidation (C8–C12)	Anti-MDR, anti-biofilm, membrane permeabilization, protease-stable, low resistance	SPPS, D-AA incorporation, fatty-acid conjugation	Ano-D4,7-C8/-C10/-C12 analogs [89]
Ultra short Synthetic AMP	D-amino acid substitution in α-helical AMP	Protease stability and selectivity, antibiofilm activity	SPPS (Fmoc/tBu chemistry)	KR-12-a5 (D-Leu6 analog) [90]
Synthetic cyclic peptide	Catalyst-free cysteine stapling using fluorobenzene linkers (ortho/meta/para)	Tunable macrocycle size, enhanced stability, controlled secondary structure, improved receptor potency	SPPS + on-resin or solution-phase cysteine stapling	Fluorobenzene-stapled melanocortin receptor agonist peptides [91]
Synthetic stapled AMP	Dual strategy: all-hydrocarbon double stapling + arginine N-glycosylation	High antimicrobial potency, proteolytic stability, and biofilm inhibition	SPPS	SLP-51 (stapled and glycosylated SAAP-148 analog) [92]
Synthetic peptoid AMP	Rational design based on CAMP charge–hydrophobicity score	Protease resistance, membrane-targeting activity, conformational flexibility, and high antimicrobial potency	Solid-phase peptoid synthesis (sub-monomer method, Rink amide resin)	Peptoid P13#1 [93]
Synthetic ultrashort peptidomimetic AMP	Tetra-peptide (Orn–Orn–Trp–Trp) conjugated to hydrophobic cinnamic acid derivative	Strong antibiofilm activity, effective against MDR Gram-positive pathogens, low cytotoxicity, and cost-effective	Solid-phase peptide synthesis (SPPS) with N-terminal cinnamic acid conjugation	(3-(4-Hydroxyphenyl)propionic)-Orn-Orn-Trp-Trp-NH_2_ [94]
Synthetic ultrashort AMP (LL-37–derived)	Rational truncation of human LL-37 using optimized screening medium; miniaturization for cost-effectiveness	Potent anti-MRSA activity, antibiofilm and biofilm-disrupting ability, low mammalian toxicity, and low resistance development	Solid-phase peptide synthesis (SPPS)	LL-37mini (derived from KR-8/KR-12 region of LL-37) [95]

### 5.3. Anti-Biofilm Mechanisms

UP-5, an ultrashort synthetic peptide synthesized by the SPPS method, has shown great antimicrobial activity against multidrug-resistant bacteria. Its antibiofilm activity is primarily driven by its electrostatically binding to negatively charged bacterial membranes, followed by membrane insertion mediated by hydrophobic residues. This interaction disrupts membrane integrity, causes pore formation, and increases permeability, thereby facilitating cell lysis and enhancing antibiotic penetration into cells embedded within biofilms. Additionally, membrane destabilization affects biofilm structural stability, leading to reduced biofilm viability at comparatively low peptide concentrations [96]. Another synthetic peptide, WLBU2, has been shown to affect the formation of biofilms by causing the bacterial cell membrane to become permeable and be destroyed, which leads to interference with early surface attachment. In addition, it acts strongly against the quorum sensing pathways at sub-inhibitory concentrations by down-regulating the important las and rhl regulatory genes [97]. The strong antibiofilm potential of synthetic peptides was also confirmed by in vivo results. The synthetic PS peptides, which possess strong bactericidal activity, also prevent biofilm development by interfering with initial cell attachment. Their strong cationic and amphipathic nature drives the whole process. In addition, they weaken mature biofilms by disrupting the extracellular polymeric matrix, which comprises carbohydrates, lipids, proteins, and extracellular DNA. This weakening of the structure results in a lessening of the biofilm’s strength, hence making it easier to remove the biomass even in drug-resistant strains of *S. aureus* and *P. aeruginosa* [98]. Another important aspect of these peptides is their ability to inhibit biofilms at high salt conditions, which speeds up the killing of both drug-sensitive and -resistant planktonic cells before mature biofilms form. Their amphipathic and cationic character, especially the N-terminal tryptophan, helps them attach to the membrane, penetrate the EPS, and eradicate the bacteria within mature biofilms [99].

### 5.4. Advantages

**Figure 5:**
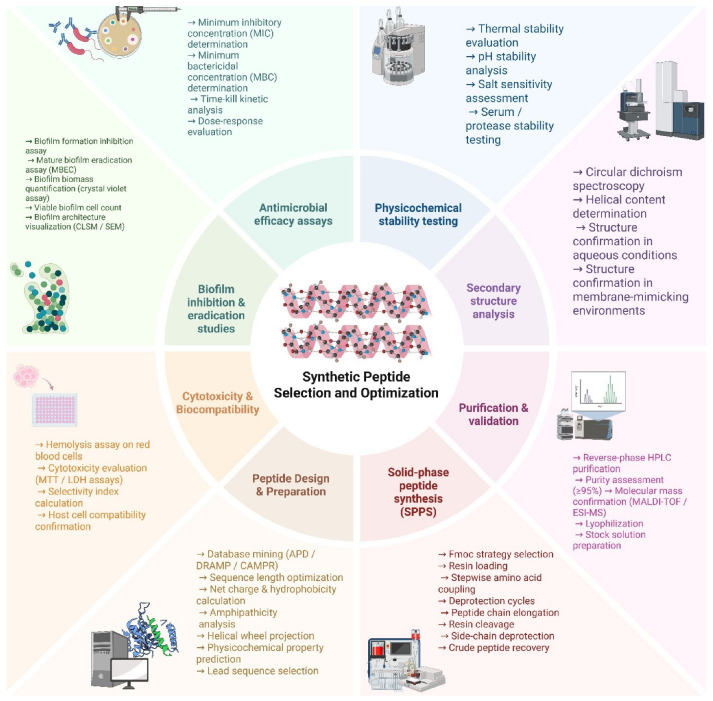
Integrated workflow for the rational design, synthesis, characterization, and biological evaluation of synthetic antimicrobial peptides.

The synthetic peptides demonstrate higher efficacy against biofilms because of their versatile design strategies (Figure 5). Solid-phase peptide synthesis (SPPS) provides a method of precise and gradual control of the choice of amino acids, leading to the highest accuracy of synthetic peptide formation. Furthermore, non-natural amino acids can be incorporated to improve the stability, potency, and resistance to enzymatic degradation of these peptides [100]. Synthetic peptides offer enhanced thermal stability, which is crucial for their biological function, even at considerably higher temperatures. Many of these compounds are designed to be resistant to proteolytic degradation, including trypsin-induced hydrolysis [101]. Moreover, the synthesis of SAMPs allows rapid identification of highly effective AMP sequences based on insights into the structure–activity relationship [102].

## 6. Engineered AMPs: Modified Peptides with Boosted Potency

**Figure 6:**
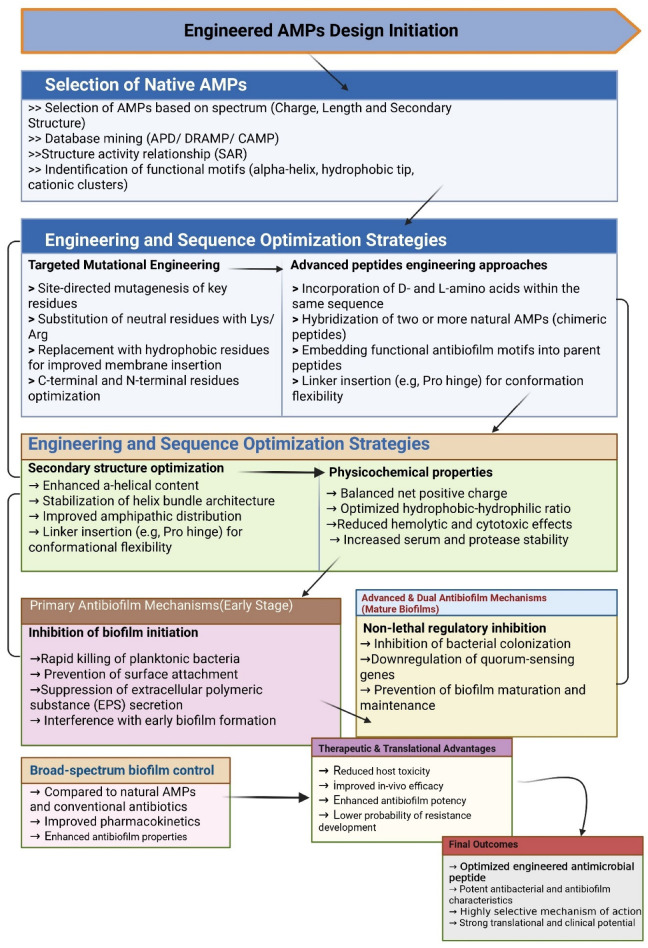
Stepwise framework of engineered antimicrobial peptides with enhanced antibacterial and antibiofilm performance.

The structural modification of engineered antimicrobial peptides (EAMPs) and their conjugation to polymers lead to the improved properties of the peptides as shown in Figure 6. The modifications boost stability, solubility, and compatibility with mammalian cells, while maintaining or even enhancing antimicrobial activity, which is exactly the aim of engineered AMPs [103]. The recent progress in the structural description, optimization, and design of delivery methods, such as their combination with nanoparticles and rational engineering, have made AMPs more powerful, stable, and selective. Utilizing two or more antimicrobial peptides in personalized medicine and combining them with current drugs can overcome the existing limitations of traditional antimicrobials [104].

### 6.1. AMP Engineering Strategies

Through the utilization of different mutagenesis techniques with distinctive mutational spectra, there has been an improvement in methods for detecting alternative protein and peptide substances with specific biological functions and preferred physicochemical characteristics [105]. Site-directed mutagenesis involves the deliberate alteration of specific amino acids at key locations, such as those participating in hydrogen bonding, charge distribution, or hydrophobic interactions. By replacing certain residues with positively charged or hydrophobic amino acids, it is possible to significantly improve the peptide’s interaction with the membrane, its selectivity, and ultimately, its overall antimicrobial effectiveness [106]. This technique has been used to systematically alter specific amino acids in the C-terminal region of pediocin PA-1 AMP to enhance its antimicrobial activity. By substituting individual residues, such as glycine at position 29, with alanine, researchers identified key sites that influence the peptide’s ability to interact with and penetrate bacterial membranes. A point mutation can mechanistically enhance peptide selectivity by changing side-chain characteristics, which include charge, polarity, and steric bulk. This change has two effects: it weakens non-target binding while improving binding accuracy [107]. The approach also revealed that combinations of mutations can optimize structural features, like the hydrophobic tip and α-helix bundle, to improve insertion into the membrane and binding to target receptors, ultimately increasing antibacterial potency [108]. In addition, enhancing charge or hydrophobicity in antimicrobial peptides (AMPs) has significantly advanced their therapeutic potential, necessitating a careful balance among toxicity, antibiofilm properties, and antibacterial efficacy [109]. The authors of [110] designed an engineered antimicrobial peptide with charge distribution and hydrophobicity deliberately optimized through the design of coiled-coil heptad repeats. This was done by tuning the heptad composition and linker length, leading to controlled helical structure, amphiphilicity, cell selectivity, and biofilm activity. Another effective method for enhancing the characteristics of native AMPs is to combine two or more natural AMPs to form a new hybrid peptide [111]. For example, B1 is a 25-amino acid chimeric peptide derived from the N-terminal region of BMAP-27 (residues 9–20) and the C-terminal segment of LL-37 (residues 17–29). It carries a net positive charge of +8, contributed by multiple lysine and arginine residues. This cationic property enables effective interaction with the negatively charged bacterial membranes of both Gram-positive and Gram-negative species [112].

### 6.2. Anti-Biofilm Mechanisms

Substantial evidence indicates that engineered AMPs can inhibit biofilm-forming microbes. Four α-helical antimicrobial peptides were rationally engineered by combining D- and L-amino acid forms within the same sequence, resulting in a >30-fold increase in antimicrobial potency. These peptides inhibit biofilm formation by killing or destabilizing bacteria precisely at the moment biofilm formation begins [113]. Another engineered AMP, Lf-KR, was rationally designed by linking two known AMPs (LfcinB6 and KR-12-a4) via a Pro hinge, a modification that does not occur naturally and is intended to enhance selectivity, stability, and function. This engineered peptide exerts antibiofilm activity primarily through membrane permeabilization and depolarization, leading to the rapid killing of biofilm-embedded cells. It also destabilizes the structural integrity of established biofilms by disrupting both outer and inner bacterial membranes [114]. Antibiofilm activity was also seen in R-FV-I16, an engineered peptide rationally designed by embedding a functional antibiofilm sequence (FV7) into the middle of an existing peptide (RI16) to replace a defective segment (RR7), creating a novel hybrid with enhanced antimicrobial and antibiofilm activity. It inhibits biofilm formation by killing bacteria through permeabilization and depolarization of outer and inner membranes, while at higher concentrations, this engineered peptide can also disrupt preformed biofilms by destabilizing cell clusters and the extracellular matrix [115]. Some EAMPs possess a dual mechanism of biofilm inhibition; this mechanism is seen in the antimicrobial peptide A24, which targets both planktonic and biofilm-embedded cells of multidrug-resistant Gram-positive bacteria. It was rationally designed and engineered using the CAMP bioinformatics tool with a random forest algorithm, based on the AP138 parent sequence. It disrupts bacterial membranes, causing leakage of cellular contents and hindering initial biofilm formation. In mature biofilms, it penetrates the matrix, activates dormant cells, produces reactive oxygen species, and kills bacteria, effectively preventing and eradicating biofilms [116]. EAMPs can also interfere with the production of extracellular polysaccharides and downregulate quorum sensing genes in *S. epidermidis*, preventing bacterial attachment and early biofilm development. They rapidly kill planktonic and biofilm-associated cells through membrane-targeted activity, forming an α-helical amphipathic structure that disrupts bacterial membranes. Additionally, it can eradicate mature biofilms and prevent biofilm formation on surfaces, as shown in catheter-coating experiments [117].

### 6.3. Advantages

EAMPs have the potential to be a substitute for conventional antibiotics. They can overcome the above limitations of toxicity, in vivo validation, and poor bioavailability, which are often seen in natural peptides [118]. EAMPs are easy to design, and general methodologies can be used to overcome numerous limitations [119]. Hybrid peptides, the new and promising engineered candidates, are formed by rationally fusing sequences from two or more different peptides whose biological activities are complementary. The idea behind this method is to combine the different features of the existing sequences or to create entirely new functions through sequence integration. The therapeutic potential of hybrid peptides is much higher than that of natural AMPs, and they have remarkable applications in the antibacterial, anticancer, and metabolic disorder fields [120]. Additionally, these peptides are economically advantageous because the polymerization techniques offer numerous benefits that play a key role in enabling the straightforward production of high-molecular-weight peptide hybrid polymers [121].

## 7. Synthetic Versus Engineered AMPs: A Direct Comparison

**Figure 7:**
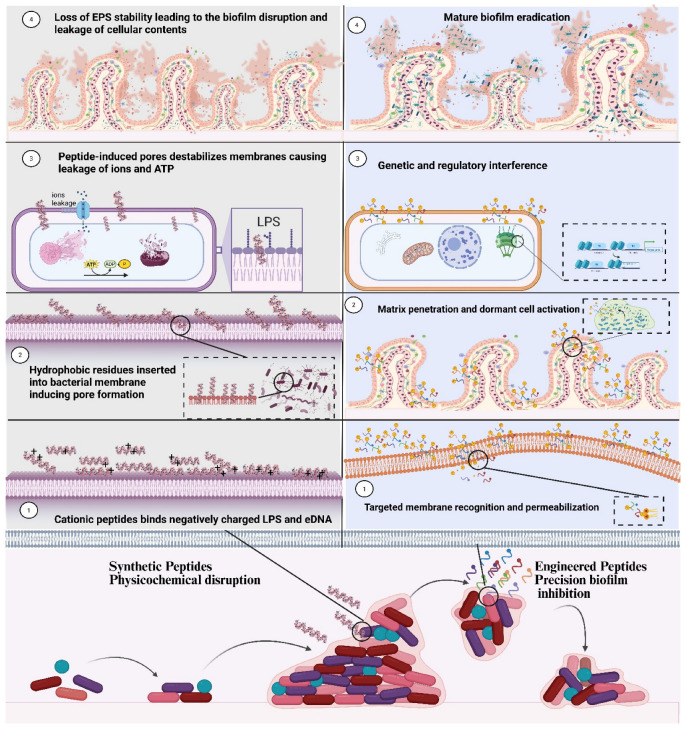
Comparative mechanism of biofilm inhibition by synthetic and engineered peptide.

Synthetic antimicrobial peptides hold great promise for the treatment of both planktonic and biofilm-based bacterial and fungal infections. They are notable for their greatly enhanced stability, activity, and resistance to proteolytic breakdown. In addition, these peptides are characterized by reduced toxicity, lower production costs, and improved structural–functional understanding, which leads to increased clinical applications [122]. In contrast, engineered peptides are the result of deliberate structural modifications to enhance function instead of just mimicking the original sequences [65]. The efficacy of these peptides in biofilm prevention (Figure 7) and elimination has been well-reported and extensively discussed in the scientific literature [123].

### 7.1. Mechanistic Comparison

The synthetic peptide [W7]KR12-KAEK inhibits the biofilms of *E. faecalis* by rupturing membranes, degrading the extracellular matrix, and changing the metabolism and gene expression. Its effects are enhanced due to the presence of a positive charge, amphipathicity, and tryptophan. However, the limitations of strain-dependent susceptibility, reduced effectiveness against mature biofilms, and the need for high concentrations for complete eradication limit its antibiofilm properties [124]. The engineered peptide PEW300, which incorporates three targeted point mutations (E9H, D17K, and T33A) designed to improve its antibacterial and antibiofilm properties, overcame these drawbacks. PEW300 primarily disrupts mature biofilm structures at low concentrations, which exposes the adhered bacteria to antimicrobial action, thereby inhibiting biofilms. It targets extracellular DNA, a key component of the biofilm matrix, while also disrupting bacterial membranes, generating intracellular reactive oxygen species, and interacting with genomic DNA. It also disperses mature biofilms by targeting extracellular DNA and reduces virulence by downregulating genes responsible for elastase, pyocyanin, pyoverdine, and alginate production [125]. One of the major contributors to strength in biofilms is quorum sensing. A type of synthetic peptide known as dipeptides (CDPs), designed and chemically modified from the natural cyclo(L-Trp-L-Ser), have been shown to block QS-controlled virulence by lowering violacein and pyocyanin levels, and by reducing biofilm formation and adhesion, likely through competitive binding to QS receptors such as CviR. They were effective at 1 mM with minimal cell toxicity or red blood cell damage. However, there are a few drawbacks, like the relatively high concentrations required and the fact that only moderate reduction is achieved in some virulence traits because of the complex QS network, with deep QS inhibition still not clearly demonstrated [126]. Engineered antimicrobial peptides utilize computational and structure-guided design to enhance antibacterial, antibiofilm, and safety profiles beyond conventional synthetic AMPs. These peptides disrupt biofilms by destabilizing the extracellular matrix, damaging membranes, and interfering with bacterial communication. Their amphipathic cationic nature enables strong interactions with biofilm components, supporting resistance-free biofilm eradication. However, reduced activity against Gram-negative biofilms, along with the absence of in vivo validation, long-term resistance studies, and multispecies biofilm evaluation, remains a key limitation in some of these peptides [127].

### 7.2. Performance Against Biofilms of Priority Pathogens

**Figure 8:**
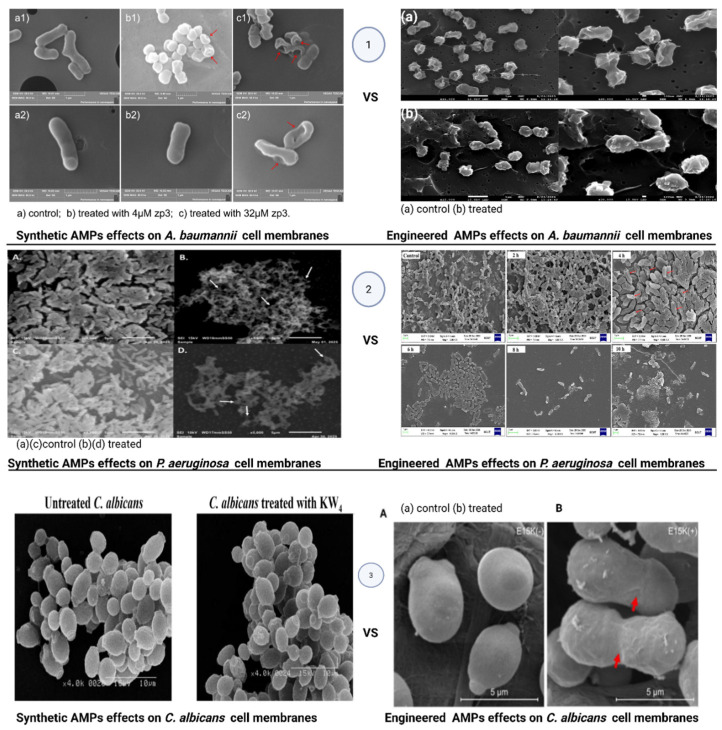
Comparative scanning electron micrographs illustrating membrane-disruptive and antibiofilm effects of synthetic versus engineered antimicrobial peptides against *Acinetobacter baumannii* [137,141], *Pseudomonas aeruginosa* [125,142], and *Candida albicans* [143,144], where untreated controls exhibit intact and smooth cell surfaces, while peptide-treated cells show progressive surface roughening, pore formation, deformation, collapse, and biofilm disintegration, with engineered peptides inducing more pronounced and time-dependent structural damage, highlighting their enhanced potency and distinct modes of action at the cellular membrane level.

Biofilms formed by *S. aureus* have many harmful biomedical effects. These biofilms result in chronic infections that are challenging to eradicate. In addition to undermining therapeutic effectiveness, biofilm formation contributes to the malfunction of medical devices and increases the overall risk of adverse clinical outcomes for patients [128]. This problem was recently addressed using engineered peptides. One such engineered peptide, mKLK, inhibited *Staphylococcus aureus* biofilms by preventing initial surface attachment, suppressing biofilm formation at sub-MIC levels, penetrating the extracellular matrix, and dispersing established biofilms through membrane disruption and interference with biofilm-regulatory signaling. Rational sequence engineering, including strategic tryptophan insertion and optimized amphipathic helicity, further enhanced its antibiofilm activity [129]. The synthetic peptides also showed strong antibiofilm activity against this pathogen, including Anoplin_k1, because they interact more strongly with bacterial membranes through increased hydrophobicity, block bacterial adhesion, and interfere with cell-to-cell signaling that controls biofilm formation. However, limitations include poor penetration into mature biofilms due to peptide size, damage to red blood cells at higher hydrophobicity, and the need for testing on different surfaces, with longer exposure times, and in living organisms [130]. *Pseudomonas aeruginosa* infections, particularly those involving biofilm formation, pose significant therapeutic challenges because of their strong intrinsic and acquired resistance to antimicrobial agents. As a result, healthcare facilities across the globe are witnessing a continuous rise in cases associated with multidrug-resistant *P. aeruginosa* [131,132]. Synthetic peptides have been recognized as a new technology for combatting biofilm formation in this bacterium. The synthetic peptide Tritrp and its derivatives prevent the development of *Pseudomonas aeruginosa* biofilms by creating holes in the cell membranes, leading to lipid flip-flop, disturbing the cytoplasmic membranes, and impeding the synthesis of DNA, RNA, and proteins. The antibiofilm activity is further enhanced by Pro substitutions and Lys or Dap modifications that increase selectivity and membrane binding [133]. A specifically engineered peptide was equally active against *P. aeruginosa* biofilms and, at 2 μmol/L concentration, destroyed over 76% of aged biofilms. However, its particular mode of action is still uncertain, unlike that of synthetic peptides [134]. *Acinetobacter baumannii* is a bacterium that easily produces strong biofilms on both living and non-living surfaces, and is thus able to survive for a long time in hospitals and outdoors. The biofilms make it very difficult to kill bacteria using antibiotics or disinfectants because they not only prevent drug entry but also allow the bacteria to evolve into less sensitive forms or to adapt to stressful conditions [135]. Synthetic peptides can damage *Acinetobacter baumannii* cell membranes, as depicted in Figure 8, increase harmful molecules inside the cells, enter the biofilm layer, stop biofilm formation, and partly remove biofilms that are already formed. This effect was observed with the synthetic peptide Octominin, in a study that was the first to demonstrate Octominin’s combined antibiofilm activity, low toxicity, and protective effect against multidrug-resistant *A. baumannii* in a zebrafish infection model, highlighting its potential for future medical use. A key limitation is its lower activity against mature biofilms, which reflects the natural resistance of established biofilm structures to peptide penetration [136]. This limitation was overcome by a rationally designed engineered peptide called HRZN, which was created using database-filtering technology (DFT) and a positional analysis (PA) database (GRAMPA). This peptide’s antibiofilm activity is mainly driven by rapid disruption and opening of the bacterial cell membrane, which quickly causes the death of *Acinetobacter baumannii* cells inside biofilms, including fully developed biofilms, but is also associated with toxicity to the host, showing that further optimization of the peptide sequence is needed [137]. Fungal biofilms represent highly organized, surface-associated communities that confer remarkable tolerance to antifungal agents and host immune defenses. Among these, *Candida albicans* is the most clinically significant species, capable of forming robust biofilms on both biotic and abiotic surfaces. Its biofilm lifestyle underlies persistent infections, therapeutic failure, and frequent recurrence, making it a critical target for next-generation antifungal strategies [138]. In *Candida* species, synthetic peptides disrupt biofilms by binding to cell wall chitin, perforating membranes, and triggering excessive ROS generation, which together destabilize biofilm architecture and kill embedded cells. These antibiofilm characteristics were seen in two chemically synthesized SAMPs, called *Mo*-CBP_3_-PepI and *Mo*-CBP_3_-PepIII. But further in vivo validation and pharmacokinetic optimization are required to fully assess the clinical translational potential of these peptides [139]. An engineered peptide, Blap-6, and Gomesin inhibit Candida biofilm formation by targeting key fungal proteins (LDM, Sap-5, NMT, DHFR), inducing structural damage, membrane disruption, and impaired cell viability [140].

### 7.3. Cytotoxicity and Safety Comparison

Synthetic α-helical antimicrobial peptides were tested for toxicity on blood cells to evaluate their suitability for systemic use. Highly helical peptides like l-19(9/B) are strongly toxic, causing membrane damage in mouse lymphocytes and human erythrocytes, as confirmed by propidium iodide uptake and SEM imaging. In contrast, modifications that reduced helix formation, such as P19(8), or changing the helical sense in d-P19(9/B), significantly lowered cytotoxicity, making them safer, though systemic use still showed limited effectiveness at higher doses in *Candida albicans*-infected mice [145]. It was also demonstrated that these peptides can selectively target bacterial cells while exhibiting minimal toxicity toward mammalian cells. Notably, although they were somewhat more active against canine red blood cells, they showed low cytotoxicity toward rat, human, and bovine cells, and minimal effects on HeLa, HaCaT, and HepG2 cell lines [146]. Conversely, the engineered leucocin A, most notably peptide 7 (WRL3, sequence: WLRAFRRLVRRLARGLRR-NH2), showed the highest selectivity for bacterial cells over mammalian cells like erythrocytes and macrophages. It also exhibits potent cytotoxicity against human HepG2 cancer cells, which may restrict its safe systemic use [147]. The engineered peptide VhTI-pep 2 was designed by grafting the bioactive sequence of pepitem onto a stable scaffold. Its advantage is its enhanced serum stability and retention of biological activity without harming host cells, meaning it shows no cytotoxicity. This is achieved by maintaining the peptide in a well-structured conformation that modulates immune cell migration rather than disrupting cell membranes [148]. Both these categories have overcome delivery and stability challenges through chemical modifications, nanoparticles, and cell-penetrating fusions, enhancing tumor targeting and bioactivity while minimizing toxicity. These strategies improve clinical potential against infections and cancer, addressing limitations of natural peptides [149].

### 7.4. Cost-Efficient and Flexible Design

Obtaining peptides from natural sources is time-consuming and labor-intensive, making it impractical for large-scale production, while chemical synthesis offers efficiency for small peptides [150]. A synthetic peptide was assembled through chemo-enzymatic peptide synthesis (CEPS) using engineered ligases and solid-phase methods. This approach proved to be cost-effective and scalable, yielding large quantities with high purity while minimizing environmental impact. Optimized fragment design and ligation conditions ensured both economic efficiency and sustainable large-scale production [151]. On the other hand, engineered peptides offer clear cost and design advantages while enabling precise sequence optimization, improved stability, and predictable biological activity. Rational engineering reduces trial-and-error synthesis, lowers production waste, and supports scalable, cost-effective manufacturing. Their modular design also allows easy functional tuning for targeted and safer therapeutic applications [152].

## 8. Gene Regulation and Mechanisms Underlying Biofilm Inhibition by Synthetic and Engineered Peptides

**Figure 9:**
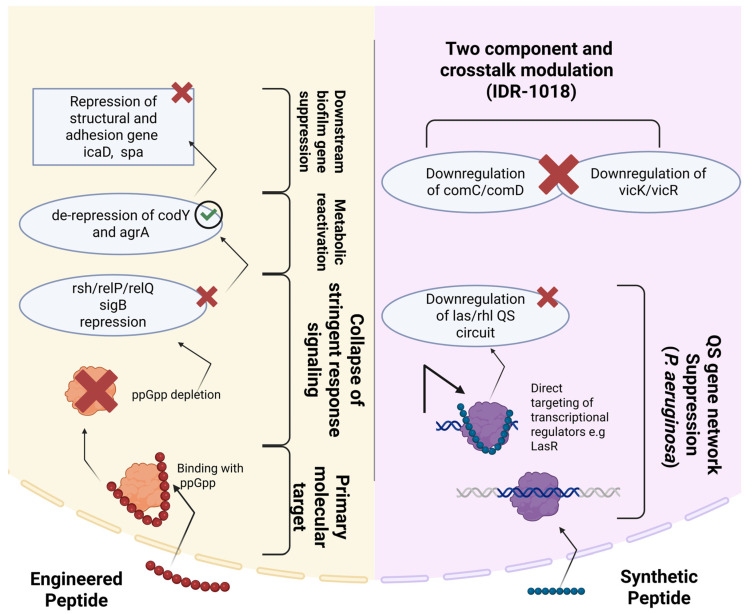
Comparative schematic of engineered and synthetic peptide-mediated antibiofilm mechanisms, illustrating global stress-response reprogramming and metabolic activation by engineered peptides versus targeted quorum sensing and regulatory pathway interference by synthetic peptides leading to biofilm destabilization.

There are numerous strategies to control bacterial biofilms by targeting their underlying genetic regulatory machinery. Use of these strategies has expanded rapidly, reflecting a shift toward gene-level intervention strategies [153]. Antimicrobial peptides (AMPs) are the most potent candidates leading to the disruption of stress-response signaling (Figure 9 (ppGpp-mediated stringent response)) and biofilm architecture, rather than direct membrane lysis [154]. 1018M is a rationally designed engineered antimicrobial peptide derived from IDR-1018. It shows remarkable activity against MRSA biofilms. Its main mechanism is the binding of the alarmone ppGpp and its subsequent depletion, which then leads to the suppression of stringent response signaling. The process of signaling suppression has its impact on the normal performance of the cell’s metabolism. It involves the reduced activity of the genes responsible for the monitoring of the stringent response (e.g., rsh/relP/relQ and sigB) and, at the same time, the activation of the genes that promote the activity of the cell’s metabolism (e.g., codY and agrA). As a result, the genes associated with biofilm formation, such as spa and icaD, are turned off (i.e., repressed), thereby creating a situation in which biofilm formation is hindered, and the bacteria’s susceptibility to the killing action of this peptide is increased [155]. As engineered peptides are specifically designed, they are rationally modified or strategically combined to optimize complementary functions. IDR-1018 acts primarily as a biofilm-regulatory peptide by modulating quorum-sensing and two-component system genes (comC/comD and vicK/vicR), leading to the destabilization of mature biofilms [156]. Furthermore, engineered Trp-containing peptides modulate bacterial signaling rather than acting solely through membrane lysis. At sub-inhibitory levels, they inhibit the development of *P. aeruginosa* biofilm through the downregulation of QS systems (las and rhl) as well as the key exopolysaccharide genes (pelA, algD, pslA). Moreover, they boost their antibiofilm action by blocking the expression of efflux pump genes (oprM, mexA, mexX) and β-lactamase genes [157]. It has also been observed that synthetic peptides like WSF, FASK, and YDVD are direct inhibitors of the LasR transcriptional activator in the *Pseudomonas aeruginosa* quorum sensing system. The peptides’ binding to LasR makes it less effective in its regulatory role, and consequently, there is a significant reduction in the expression of biofilm-related genes such as algC, pslA, and pelA. The reduction in gene expression results in decreased production of extracellular polysaccharides, poorer development of biofilms, and even less growth of free bacterial cells at the higher concentrations of the peptides [158]. Synthetic macrocyclic peptides also play an important role in modulating the accessory gene regulator (agr) quorum sensing system of *Staphylococcus epidermidis*. This is demonstrated by the fact that, whether acting as agonists or antagonists, these substances can superactivate or completely block agr receptor signaling, which ultimately leads to a remarkable decrease in biofilm formation on non-biological surfaces. Small structure-specific changes in residues make it possible to toggle between activating and inhibiting, and surprisingly, at sub-activating levels, certain peptides have a reducing effect on biofilm formation, which reveals a gene-regulatory mechanism that disrupts the QS-controlled virulence and biofilm-associated genes [159].

## 9. Delivery Strategies for Synthetic Versus Engineered AMPs for Boosted Activities

**Figure 10:**
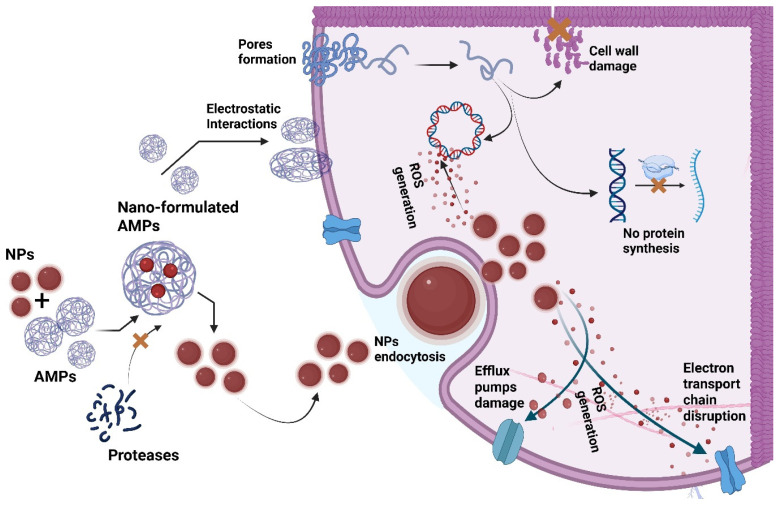
Schematic illustration of nano-enabled antimicrobial peptide (AMP) delivery, highlighting enhanced stability, cellular uptake, and targeted bacterial interactions.

Advanced delivery techniques fully exploit the effects of AMP by protecting peptides from enzyme degradation and making them stable in physiological environments. These methods not only deal with the drawback of bacterial sensitivity but also increase antimicrobial action through better targeting, as shown in Figure 10. The most significant point is that the optimized delivery method not only minimizes the toxicity to the host cells but also enables the therapeutic agent to be more effective [160]. The chemical conjugation of synthetic antimicrobial peptides to aluminum oxide nanoparticles resulted in hybrids of peptides and nanoparticles, which served as a sophisticated delivery system. This delivery system was not only responsible for the increased longevity of the peptides but also provided the peptides with better interactivity with the bacterial cells and biofilms. The nanoparticles, as a way of delivery, increased the antimicrobial and antibiofilm activities at even lower concentrations than those of peptides alone [161]. Nanoparticles were also used as a method of delivery by encasing the synthetic peptide Octominin in chitosan-carboxymethyl chitosan (CNPs) through an ionotropic gelation process. This also resulted in the stability of the peptide, controlled release in two phases, and enhanced antimicrobial and antibiofilm activity. In addition, this approach to delivery reduced the toxicity of the free Octominin, thereby demonstrating a safety margin and a more effective therapeutic potential [162]. Conversely, PLGA nanoparticles mixed with chitosan and PVA have been used in the creation of peptides to entrap them in nano-embedded microparticles (NEMs). Owing to this delivery method, the peptide was kept stable, its permeation through the mucus was assisted, and its release in the lung was made continuous. Thus, it made biofilm inhibition easier and prolonged the period of antimicrobial activity [163].

An engineered amphiphilic cationic peptide named AP1 was engineered with self-assembly and a disulfide bridge. The resulting injectable hydrogel works as a drug delivery system, giving prolonged antibacterial activity, preventing the formation of biofilms, and coating surfaces of medical devices. The hydrogel delivery method allows the antimicrobial action to be concentrated in the desired area while maintaining the compatibility of mammalian cells [164]. Hydrogels made from engineered antimicrobial peptides (EAMPs) have been made with precise amounts of hydrophobic and cationic amino acids. As well as being biocompatible, these peptide hydrogels can also serve as continual drug delivery systems. Furthermore, they keep the bacterial biofilms suppressed and help the body to recover by healing wounds, enhancing angiogenesis, and reducing inflammation [165]. The hydrogels made from synthetic peptides not only are delivery platforms, but also create a self-assembled 3D network that stabilizes and localizes the nanoparticles of silver or gold that were incorporated. They enable the bacteria to be killed more effectively and faster by having the nanoparticles in proximity to them, but at the same time, they prevent the active agents from diffusing or degrading quickly, thereby ensuring the antimicrobial action is effective and lasting [166]. Synthetic peptide-based hydrogels are widely valued as biomaterials due to their versatile protective and supportive roles [167]. These structures physically entrap therapeutic molecules through hydrophobic interactions, π–π stacking, and confinement within the fibrillar matrix. Porous and flexible hydrogels based on synthetic peptides are, therefore, non-toxic and capable of continuous and regulated release of drugs that are either hydrophobic or hydrophilic [168]. Hydrogels containing chitosan and oxidized dextran were used to form a hydrophilic matrix that served as a multifunctional delivery platform instead of a structural peptide gel. The HHC10 synthetic antimicrobial peptide that is part of the hydrogel network is responsible for breaking down the bacterial membranes, while the incorporated photosensitizer produces reactive oxygen species through light exposure, thus combining two effective methods of killing bacteria. Due to the pH-dependent characteristic of the matrix, both agents can be released in a controlled manner and sustained over time in the bacteria-infested wound area [169]. The antimicrobial peptide LL17–32 is a synthetic chemical compound formed by the human cathelicidin LL-37 short active fragment, and has been used as a therapeutic agent in medicine. This peptide does not create a self-assembled structure but rather attaches to the already existing phospholipid liposomes made from either neutral DOPC or negatively charged soya lecithin. These liposomal vesicles work like nanoscale carriers that not only hold the peptide but also control its interactions with bacterial membranes. The lipid–peptide structure boosts the antibacterial activity against Porphyromonas gingivalis while at the same time minimizing the toxicity to host gingival cells [170]. The engineered synthetic antimicrobial peptides sequences were rationally designed and chemically synthesized to impart cell-penetrating and antimicrobial properties. Instead of acting as the carrier themselves, these peptides are covalently attached to the liposomal surfaces, causing a virus-like nanostructure to be created with a very high local positive charge density. This structure increases the electrostatic interactions with negatively charged bacterial membranes and biofilms, leading to more efficient membrane disruption and higher antibacterial activity. The liposomal delivery system, on the other hand, controls the amount of peptide that is exposed, thus maintaining potent antimicrobial activity and at the same time significantly reducing the cytotoxic effects on mammalian cells [171].

## 10. Emerging Directions in Peptide-Based Biofilm Therapies

**Figure 11:**
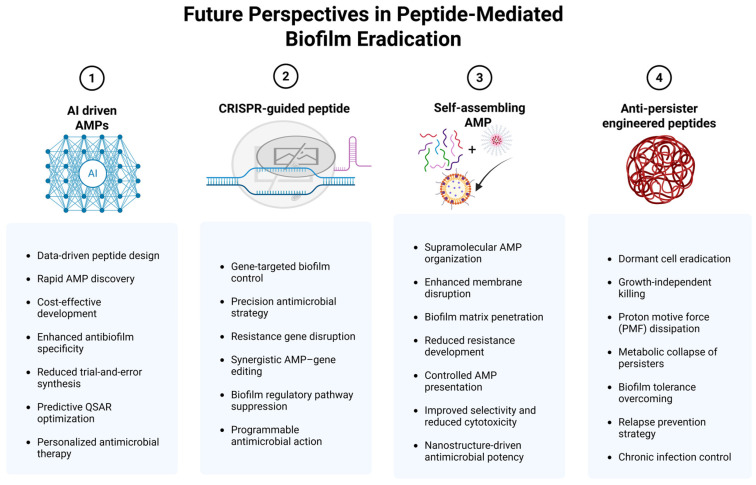
Overview of emerging peptide-based strategies for biofilm eradication, including AI-designed AMPs, CRISPR-guided peptides, self-assembling systems, and anti-persister approaches.

The successful clinical translation of antimicrobial peptides is dependent on continuous long-term research development, as depicted in Figure 11, and the collaboration of academic research institutions, clinical practitioners, and pharmaceutical development sectors in an interdisciplinary manner [172]. Recently, antimicrobial peptides have been developed as a promising alternative to conventional antibiotics due to the worldwide increase in the rate of drug-resistant infections. Besides experimental screening platforms, computational modeling and virtual screening have become necessary methods during the early stages of drug development. These in silico techniques enable the exploration of new molecular areas that are largely inaccessible through conventional experimental pipelines [173].

### 10.1. AI-Driven AMP Generation

Artificial intelligence played a central role in designing these peptides. The explainable AI-driven model discovered similarities within the antimicrobial and antibiofilm peptide classes that were already known, and then used this insight to come up with new sequences that had better structural and functional properties. Since the AI system did the computational pre-screening of peptide candidates, only those with predicted high therapeutic potential were taken for experimental synthesis and validation, which consequently resulted in a reduction in the expensive trial-and-error synthesis [174,175]. Synthetic peptides that were designed with deep QSAR were generated by means of a machine learning approach and compared to the well-known reference antibiofilm peptide IDR-1018. The newly obtained peptides exhibited a higher bactericidal activity against pathogens like *Staphylococcus aureus* and a better ability to prevent and disrupt bacterial biofilms. Their main mechanism of action is the destruction of bacterial cell membranes and the inhibition of protective biofilm layer formation, which makes infections easier to control than treatment with conventional antibiotics [176]. The use of AI-engineered peptides is revolutionizing the drug discovery process as they facilitate the swift design, optimization, and prediction of peptide therapeutics with very high precision and personalized treatment potential. The primary advantages of using these peptides are the shorter development periods, lower experimental costs, and improved targeting achieved through the application of data-driven design methods. Nonetheless, there are significant difficulties regarding the model’s lack of transparency, the need for experimental validation, and regulatory acceptance that need to be addressed and resolved before these peptides can be used safely and reliably in clinical practice [177]. However, recently, AI has played a transformative role in AMP research, with machine learning, deep learning, transfer learning, and QSAR-based models being used to forecast antimicrobials’ power, minimal inhibitory concentration, safety, blood cell destruction, immune response, and protein enzyme resistance. These methods allow researchers to minimize their need for expensive laboratory testing while speeding up the process of improving peptides [178,179]. AI serves as a preclinical screening tool that assesses peptide stability and permeability, safety, and biological activity to identify candidates who have better chances of achieving in vivo success before conducting expensive animal or human testing. The process enables direct movement from computational design work to experimental validation efforts. The system faces limitations because its data shows irregular patterns, testing methods produce different results, data distribution shows unevenness, and its sequence matching system produces inaccurate results. This research needs robust validation frameworks together with standardized data integration systems to develop practical applications for real-world testing [180,181].

### 10.2. CRISPR-Guided Peptide Evolution

CRISPR-guided antimicrobial peptides (AMPs) enable the precise targeting of bacterial genes, enhancing the effectiveness of biofilm inhibition strategies. CRISPR-Cas systems, which were initially an immune mechanism in prokaryotes, are now considered a powerful means for accurate and complete removal of particular genes, such as those responsible for antimicrobial resistance. Their main areas of application include gene editing, disease modeling, and biofilms [182,183]. CRISPR technology has been used to guide peptides in the inhibition of biofilms in a resistant strain of *Escherichia coli*. CRISPR/Cas9-mediated deletion of the kduD gene revealed a target that is necessary for the above-mentioned characteristics. The antimicrobial peptide CRAMP-34 was effective in eradicating the formed biofilms, inhibiting bacterial movement, and lessening the expression of the genes responsible for biofilm formation [184].

### 10.3. Self-Assembling AMP Nanofibers

Self-assembling peptides (SAPs) are organized nanostructures created by molecular interactions with unique physicochemical properties. In addition, their high biological activity and low toxicity give them a chance as drug delivery agents, antimicrobials, antibiofilm, tissue engineering, and regenerative medicine applications. Antimicrobial SAPs are a viable option in the fight against infections that come with a risk of resistance because they are less harmful than traditional antibiotics [185]. Self-assembling antimicrobial peptides (AMPs) are capable of organizing into helical nanofibers, with molecular chirality being a crucial factor not only for stability but also for antimicrobial potency. Heterochiral nanofibers (DL configuration) can build up more stable structures that can efficiently tear apart bacterial membranes, leading to increased effectiveness against both Gram-positive and Gram-negative bacteria [186]. In the fight against microbial resistance and biofilm interaction, three self-assembling gemini peptide amphiphiles with varying spacers (Arg, Lys, His) were synthesized and tested. Out of these, 12-(Arg)_4_-12 produced tiny rods that quickly separate to break up bacterial membranes, demonstrating the most potent antimicrobial and antibiofilm action against a wide range of species [187]. The synthetic nine-amino-acid peptides, with Peptide K6 leading the group, are designed for highly effective antibacterial and antibiofilm applications. These peptides can form nanostructured micelles, which facilitate bacterial membrane disruption while also limiting the development of resistance. Peptide K6 has demonstrated effectiveness against Pseudomonas aeruginosa and Staphylococcus aureus in vitro and in vivo, indicating potential as a new antimicrobial therapy [188]. Due to their tendency to damage mammalian cells during the process of action against bacteria, the antimicrobial peptides have restricted their application in therapy. However, the mixture of a natural AMP like melittin and a synthetic peptide, which forms a β-sheet, yields the nanofibers that are prepared by self-assembly, presenting the AMP in a controlled manner, thereby creating the selective disruption of bacterial membranes and biofilms while causing no harm to mammalian cells [189].

### 10.4. Anti-Persister Engineered Peptides

**Figure 12:**
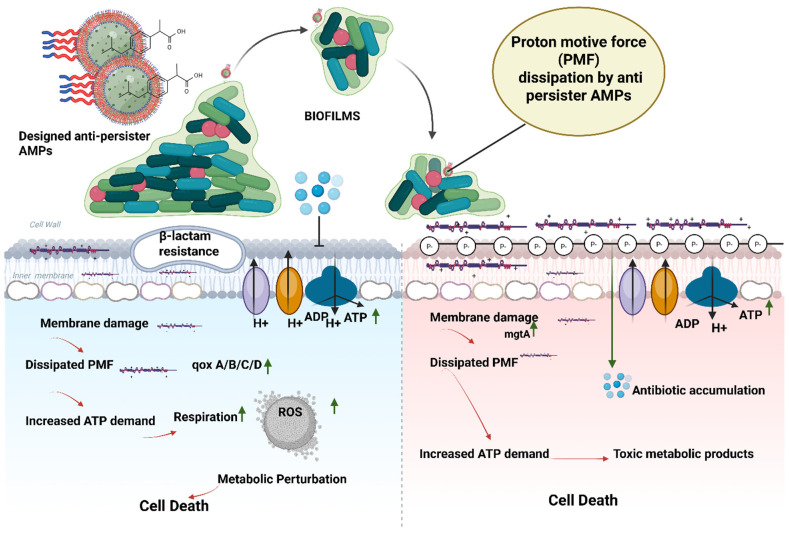
Designed anti-persister antimicrobial peptides eradicate biofilm-embedded and persister cells by targeting the bacterial cytoplasmic membrane and collapsing the proton motive force (PMF).

Persister cells are metabolically inactive but genetically susceptible bacterial subpopulations that persist in the presence of antibiotics and other hostile conditions. They are the main reason for chronic infections, relapse, and failure of antimicrobials because of their survival under treatment and subsequent growth after the stress is removed [190]. Antimicrobial peptides are considered efficient substitutes for persister cell targeting mainly due to their mode of action, which does not depend on the existence of active bacterial growth, being membrane-directed. Thus, they can exterminate the dormant and antibiotic-resistant bacteria in biofilms [191]. Latest advancements in medical science have led to the engineering of sequence-defined antimicrobial peptides as a novel strategy to target bacterial persister cells. One such engineered peptide-like molecule, known as TM5, effectively eliminates dormant persisters and disrupts established biofilms in both Gram-positive and Gram-negative bacteria [192]. LI14, an engineered antimicrobial peptide, was developed with the help of database filter technology to fight against multidrug-resistant bacteria. It has demonstrated quick bacterial killing, can penetrate biofilms, and kills “sleeping” persister cells, and is also tolerant and stable under different treatments. In terms of mechanism, it directly attacks the membranes of persister bacteria and destroys the proton motive force, as depicted in Figure 12, thereby making it impossible for the bacteria to produce energy and carry out vital functions. It also causes the bacteria’s metabolism to go out of control and breaks down the biofilm, which allows the antibiotic-tolerant organisms that are in a dormant state to be exterminated quickly [193].

## 11. Clinical Development Status and Translational Barriers

Peptides are important in clinical settings because they can accurately bind to specific targets, have flexible structures, and generally have safe profiles. The increasing number of approved peptide-based drugs, along with many others in development, shows that peptides are now a vital part of modern medicine. Their therapeutic role now extends across multiple clinical areas, including metabolic disorders, cardiovascular diseases, oncology, and infectious conditions, reflecting their growing relevance in translational and precision medicine [194,195]. Many synthetic peptides have advanced to late-stage clinical trials or obtained regulatory approval because their scalable synthesis and predictable pharmacodynamic properties enable their use in metabolic, oncologic, and infectious disease treatment [196]. For instance, Pexiganan has proven to be effective as a synthetic magainin analog that treats diabetic foot infections because it achieved Phase III testing while showing strong effectiveness against biofilm-associated pathogens through its topical antimicrobial properties. The treatment shows only minor improvements over standard treatments when used by itself, which demonstrates the need for dual-AMP combination treatments to improve treatment results for patients who have complex polymicrobial diabetic foot infections [197].

Similarly, the engineered antimicrobial peptides IK-12 and its derivatives IP-12 and WP-12 show specific antibacterial activity against drug-resistant pathogens while demonstrating the potential to treat bacterial endophthalmitis. The long-term safety and efficacy of these treatments in human patients needs to be determined through research [198]. Brilacidin, a synthetic defensin-mimetic peptide, has demonstrated strong effectiveness against multidrug-resistant pathogens, including *N. gonorrhoeae* and *S. aureus* and various fungi, including *Aspergillus fumigatus* and *Cryptococcus* species. The drug demonstrates its effectiveness in treating cases that conventional antibiotics cannot handle because it successfully treats infections that occur inside epithelial cells and biofilms while showing minimal toxicity to human cells [199]. This means that both synthetic and engineered peptides can be a possible approach that can be employed in topical or localized settings rather than systemic therapy. However, some major concerns still limit their systemic clinical approval despite promising in vitro activity [200]. Peptide hemolysis acts as a fundamental constraint because compounds that improve antimicrobial effectiveness through their high charge and hydrophobic properties also cause damage to mammalian cell membranes. After all, scientists have not yet discovered any structural guidelines that accurately forecast this toxic effect.

However, the combination of computational modeling and AI-driven design methodologies enables researchers to forecast membrane selectivity through in silico methods, which allow them to optimize sequences for reduced hemolysis while maintaining antimicrobial effectiveness [201]. Cytotoxicity also limits peptide therapeutics because their non-specific interactions at higher concentrations with host cell membranes and intracellular components lead to unintended cell damage, which results in a restricted therapeutic window [202]. Translational research for peptide therapeutics encounters further multiple obstacles, including proteolytic degradation that causes serum instability and rapid renal clearance, leading to reduced systemic half-life and the risk of immunogenicity that comes with multiple treatment sessions. The production of peptides at an industrial scale incurs high expenses, which creates difficulties during regulatory processes because peptides exist in a classification that lies between small molecules and biologics. The FDA and EMA have approved many peptides for specific medical uses, yet only some peptide candidates receive approval because their clinical success depends on safety, stability, efficacy, and manufacturing feasibility [203,204,205].

## 12. Challenges, Limitations, and Future Perspectives

Biofilms represent one of the most resilient microbial states across clinical, industrial, and environmental settings and are a major cause of chronic and device-associated infections. Their complex architecture, metabolic heterogeneity, and tightly regulated stress-response networks severely limit the efficacy of conventional, growth-dependent antibiotics. Antimicrobial peptides (AMPs) are a great option for replacing antibiotics. They are effective against bacteria that are resistant to antibiotics because they kill the bacteria, destabilize the extracellular matrix, and affect the stress signaling of the bacteria in the biofilm. The transition from natural to synthetic and engineered AMPs indicates a transition to rational peptide design, where synthetic peptides are chosen because they are chemically stable and simple, and can be produced on a large scale, while engineered peptides are chosen because they are biologically precise, have a reduced host toxicity, and can target and control the biofilm pathways. It is therefore necessary to carry out a comparative evaluation of these complementary strategies for progressing peptide-based antibiofilm therapies to clinical use.

Both peptide classes, however, encounter hurdles in their transformation from research to clinical use. It is possible for synthetic peptides to show non-specific activity towards membranes, and, thus, their application to host cells may limit their long-term effectiveness against fully developed or mixed bacterial biofilms. On the other hand, the use of selectively engineered peptides is often restricted by the intricacy of the design, the increased need for production and costs, and the limitations in scaling up. Furthermore, both methods are impacted by pharmacokinetic factors, such as fast elimination from the body, limited penetration into tissues, and sensitivity to enzymatic breakdown, along with regulatory and manufacturing issues.

The future of peptide-based biofilm control will rely on the combination of chemical durability with biological suppression. The use of AI, structure-guided design, and machine learning is believed to speed up the process of peptide optimization while cutting down on the time for chemical development. The hybrid peptide designs, along with the delivery platforms developed thereafter, such as nanoparticles, hydrogels, and self-assembling systems, will be vital in enhancing the stability, localization, and prolonged activity of the peptide in the biofilms. The targeting of specific processes in biofilms, such as quorum sensing and persister cell metabolism, is a promising route for the prevention of relapse and chronic infection. Rationally designed peptides are therefore becoming the main candidates for the next generation of antibiofilm pharmaceutics.

## 13. Conclusions

Altogether, AMPs display substantial ability in fighting AMR and treating biofilm-associated infections. Unlike various antibiotics, AMPs usually employ swift mechanisms of action, such as impeding cellular cascades, membrane perturbation, and immune modulation, lessening the selection pressure that is essential for the development of resistance and persister cells. Likewise, penetration into the protective extracellular matrix that develops during biofilm formation may be attained through improved potency and specificity of engineered AMPs. Furthermore, synthetic AMPs can restrict biofilm formation at initial stages, as they can intervene in biofilm and quorum sensing cascades. Recent advancements in the field reveal a beneficial and practical next-generation therapeutics intended for controlling biofilm-mediated infections and tackling the global health threat of AMR. However, more research is needed into pharmacokinetics, delivery barriers, and regulatory hurdles.

## Figures and Tables

**Table 1:** Bacterial-based antimicrobial peptides (AMPs), their sources, structural characteristics, and key limitations influencing antibiofilm efficacy.

AMP Type	Source/Example	Structural Features	Limitations for Antibiofilm Activity	References
Bacteriocin (Plantaricin)	*Lactiplantibacillus plantarum* Z057 (Plantaricin Z057)	Ultra-low MW (1272.6 Da), short linear peptide, glycine-rich, hydrophobic residues (Ile, Leu, Tyr), cationic	Reduced activity at neutral/alkaline pH, protease sensitivity, mechanism not fully elucidated	[35]
Lantibiotic (Class I AMP)	Nisin Z	Ribosomally synthesized, lanthionine-containing, cationic, membrane-active, chelator-enhanced permeability	Ineffective against mature biofilms, limited activity in polymicrobial biofilms	[36]
Bacteriocin PPB/PB (pore-forming AMP)	*Bacillus subtilis* endophyte (MK733983)	Proteinaceous peptide, membrane-active, pore-forming, barrel-stave mechanism, phospholipid-interactive	Reduced efficacy on mature biofilms, dose-dependent response, strain-specific tolerance, and incomplete eradication	[37]
Bacteriocins (Enterocins, Class II)	*Enterococcus faecium* FM43, FM50, GM-1, Enterocin A, Enterocin B	Proteinaceous peptides, pH-stable	Reduced activity after 24 h, partial (not universal) biofilm destruction	[38]
Bacteriocin (Class II, Leucocin)	*Leuconostoc lactis*	Ribosomal peptide, proteinaceous, cationic	Limited data on long-term biofilm prevention, primarily tested in vitro	[39]
Bacteriocin (BM173, Class II)	*E. coli*-expressed BM173	Proteinaceous peptide, heat-stable, pH-stable	Primarily in vitro data, long-term biofilm prevention are untested	[40]
Bacteriocin (HW01, Class II)	*Pediococcus acidilactici*	Heat-stable, membrane-active, non-lanthionine, cationic, virulence factor production (pyocyanin, rhamnolipid, protease)	Limited effect on planktonic growth	[41]
Bacteriocins (synergistic)	Garvicin KS + Micrococcin P1	Garvicin KS: multi-peptide, protein synthesis inhibitor	Biofilm persistence due to dormancy/persister cells, poor P1 solubility	[42]
Bacteriocin (low MW, class II)	*L. plantarum* subsp. *argentoratensis* SJ33	Low molecular weight, thermostable, acid-tolerant	Low effects on pre-existing mature biofilms	[43]
Bacteriocin (DF01)	*Lactobacillus brevis* DF01	Low molecular weight, thermostable, acid-tolerant	Ineffective on established/mature biofilms, post-treatment does not disrupt pre-formed biofilms	[44]
Peptide antibiotic (actinomycete-derived)	INA 5812-A/C (*Streptomyces roseoflavus* INA-5812)	Cyclic lipopeptide, glycopeptide-like, non-standard amino acids, arabinose moiety, calcium-dependent	Reduced activity against Gram-negative biofilms	[45]

**Table 2:** Fungal-based antimicrobial peptides (AMPs), their sources, structural characteristics, and key limitations influencing antibiofilm efficacy.

AMP Type	Source/Example	Structural Features	Limitations for Antibiofilm Activity	References
Fungal defensin-derived AMP	Plectasin (*Pseudoplectania nigrella*)	Cysteine-rich, β-sheet fold, Lipid II–binding	Limited biofilm matrix penetration, reduced activity in mature biofilms, mainly Gram-positive spectrum	[51]
Peptaibol (fungal AMP)	Emericellipsin A (*Emericellopsis* sp.)	Short linear peptide, non-ribosomal, Aib-rich, α-helical, amphipathic	Weak inhibition in Gram-negative biofilms, higher doses required for mature biofilms	[52]
Putative fungal AMP/peptaibol-rich fraction	*Trichoderma asperelloides* (extract, HMW fraction)	High-molecular-weight, proteinaceous, peptaibol-associated	Active component not fully identified, mechanism not fully resolved, mixture of compounds	[53]
Putative NRPS-derived AMPs/peptaibol-associated metabolites	*Trichoderma afroharzianum* (CSEAR12, CSE10BR1 extracts)	Non-ribosomal peptides, peptaibol-like	Active component not fully isolated, strain-specific antibiofilm activity	[54]
Astucin	*Aspergillus tubingensis* A01 (soil isolate)	92 amino acids, ~10.1 kDa polypeptide, non-ribosomal-like, hypothetical protein homolog (OJI81679.1)	Narrow spectrum tested, mechanism not fully resolved, activity mainly shown against staphylococci	[55]

## Data Availability

No new data were created or analyzed in this study.

## Abbreviations

AMR	Antimicrobial Resistance.
MDR	Multi-Drug Resistant.
EPS	Extracellular Polymeric Substances.
SPPS	Solid-Phase Peptide Synthesis.
ROS	Reactive Oxygen Species.
SAMPs	Synthetic Antimicrobial Peptides.
EAMPs	Engineered Antimicrobial Peptides.
